# Evidence for an amphibian sixth digit

**DOI:** 10.1186/s40851-015-0019-y

**Published:** 2015-06-15

**Authors:** Shinichi Hayashi, Takuya Kobayashi, Tohru Yano, Namiko Kamiyama, Shiro Egawa, Ryohei Seki, Kazuki Takizawa, Masataka Okabe, Hitoshi Yokoyama, Koji Tamura

**Affiliations:** Department of Developmental Biology and Neurosciences, Graduate School of Life Sciences, Tohoku University, Sendai, 980-8578 Japan; Department of Anatomy, The Jikei University School of Medicine, Tokyo, 105-8461 Japan; Mammalian Genetics Laboratory, Genetic Strains Research Center, National Institute of Genetics, 1111 Yata, Mishima, Shizuoka 411-8540 Japan; Department of Biochemistry and Molecular Biology, Faculty of Agriculture and Life Science, Hirosaki University, Hirosaki, 036-8561 Japan

**Keywords:** Limb, Digit, Pentadactyly, Amphibian, *Xenopus*

## Abstract

**Introduction:**

Despite the great diversity in digit morphology reflecting the adaptation of tetrapods to their lifestyle, the number of digits in extant tetrapod species is conservatively stabilized at five or less, which is known as the pentadactyl constraint.

**Results:**

We found that an anuran amphibian species, *Xenopus tropicalis* (western clawed frog), has a clawed protrusion anteroventral to digit I on the foot. To identify the nature of the anterior-most clawed protrusion, we examined its morphology, tissue composition, development, and gene expression. We demonstrated that the protrusion in the *X. tropicalis* hindlimb is the sixth digit, as is evident from anatomical features, development, and molecular marker expression.

**Conclusion:**

Identification of the sixth digit in the *X. tropicalis* hindlimb strongly suggests that the prehallux in other *Xenopus* species with similar morphology and at the same position as the sixth digit is also a vestigial digit. We propose here that the prehallux seen in various species of amphibians generally represents a rudimentary sixth digit.

**Electronic supplementary material:**

The online version of this article (doi:10.1186/s40851-015-0019-y) contains supplementary material, which is available to authorized users.

## Introduction

Pentadactyl limb evolution in tetrapods is an unsolved mystery in evolutionary biology. In stem-tetrapods, ancient species likely evolved polydactyl limbs from paired fins in sarcopterygians [1, 2]. Eventually, limbs stabilized in the pentadactyl state, and extant tetrapods are known to have five or less digits. Although polydactyl mutants have been reported in several amniotes species [3–5], no known extant species naturally exhibits polydactyly [5–8], for several possible reasons, including the fact that mutations that result in polydactyly also cause lethality or weakness, resulting in indirect negative selection [5]. In any case, the concept of a pentadactyl constraint is broadly accepted [7, 5], and developmental mechanisms for maintenance of the constraint have been proposed [9–11].

Some tetrapod species, however, have developed additional digit-like structures, apparently avoiding this constraint. Moles have a falciform bone at the anterior basal of the autopod in the forelimb that is specialized for digging soil [12]. Giant pandas have adapted to an herbivorous diet, and a pseudo-thumb enables their hands to manipulate plant stems [13, 14]. Elephants also have a digit-like structure derived from the sesamoid to support body weight [15].

Despite their digit-like structures and functions, however, these exceptional features are not identified as digits, but rather as specialized sesamoids or mesopodial elements. This seems to be because the basic tetrapod limb has traditionally been thought to include only five digits (pentadactyl constraint). However, the tetrapod limb was primitively polydactyl, and the pentadactyl state was a later stabilization [16]. It has been shown that the early stem-tetrapods *Acanthostega* and *Ichthyostega* had eight digits in the forelimb and seven digits in the hindlimb, respectively [17]. It is thought that both the forelimb and hindlimb of *Tulerpeton*, another early stem-tetrapod, had six digits [18]. Modern amphibians generally have four digits in the forelimb and five digits in the hindlimb, and many species of anuran amphibians have a prepollex (forelimb) and a prehallux (hindlimb), tiny skeletal elements adjacent to the anterior-most digit in the autopod [19, 20]. The prepollex and prehallux are usually classified into special forms of mesopodial elements, but whether the prepollex and prehallux in anurans are vestigial digits has remained controversial [5, 20, 21] (If the prehallux in anurans is a vestigial digit, it would indicate that the hindlimb has six digits.). These arguments have been mainly provided using evidence from comparative anatomy of adult amphibian limbs, and identification of the structure from ontogenetic/embryological data will give new insights into the argument. Indeed, the possibility that the prepollex/prehallux are digits has been addressed from a ontogenetic perspective (see [5] and references therein).

Here, we report for the first time that an extant species of anuran amphibians, *Xenopus tropicalis*, exhibits hexadactyly in the hindlimb. We also suggest that the prehallux present in other members of the *Xenopus* genus represents a rudimentary sixth digit and that the prehallux in amphibian hindlimbs generally represents a sixth digit.

## Materials and methods

### Ethical treatment of animals and animal husbandry

The law in Japan (Act on Welfare and Management of Animals) exempts experiments using amphibians from the requirement for IRB approval. Nonetheless, all surgery in this study was performed under anesthesia, and efforts were made to minimize suffering. Nigerian A (N9-1) and Ivory Coast lines of *X. tropicalis* were provided by National Bio-Resource Project at the University of Tokyo and Hiroshima University, respectively. *X. laevis* frogs were purchased from local suppliers, Hamamatsu Seibutsu Kyouzai and Watanabe Zoushoku in Japan. *X. borealis* frogs were provided by Dr. Ariizumi’s laboratory in Tamagawa University. *X. tropicalis* tadpoles were reared at 25 °C in dechlorinated tap water, and the staging methods used for *X. laevis* according to Nieuwkoop and Faber [22] were adopted for *X. tropicalis* and *X. borealis*. The tadpoles were fed powdered barley grass (Odani Kokufun Co., Ltd., Kochi, Japan). At stage 58, feeding was stopped until metamorphosis was completed. Thereafter, the froglets were fed Tubifex worms every other day.

### Cartilage and bone staining

Alcian blue staining for cartilage and alizarin red staining for bone were performed as previously reported for each of the animals (froglets and tadpoles of *X. tropicalis* [23] and *X. laevis* [24]). For 3D reconstruction, ossified elements (alizarin red staining) in the hindlimb were scanned using a fluorescent confocal microscope and reconstructed by Amira (Maxnet) following the manufacturer’s instructions.

### Alcian blue and elastica van gieson staining

The hindlimbs were fixed with Bouin’s fixative (9 % formaldehyde, 5 % acetic acid and 75 % saturated picric acid) at room temperature (RT) overnight. The hindlimbs were then washed with 70 % ethanol-saturated lithium carbonate for three days followed by dehydrating with 100 % ethanol. The hindlimbs were permeated with xylene at RT once, xylene/paraplast (1:1, Leica) at 45 °C once, and paraplast at 60 °C twice, and then embedded into paraplast at RT until solidification. The blocks were sectioned at 10 μm in thickness and stuck on slide glasses. The slide glasses were immersed in xylene for 20 min and then quickly in 100 % and 70 % ethanol. The sections were stained with resorcin-fuchsin solution (Muto Pure Chemicals, 40321) at RT for 30 min. After washing the sections with 100 % ethanol and water quickly, we stained them with Weigert’s hematoxylin solution (Muto Pure Chemicals, 4034–2) at RT for 5 min. The sections were next washed with water and then immersed in 1 % HCl/70 % ethanol for 10 s. After washing the sections again with water, we stained them with alcian blue solution (1 % Alcian blue 8GX, 3 % acetic acid) at RT for 15 min. The sections were washed again with water and then stained with van Gieson’s solution (saturated picric acid/1 % acid fuchsin solution, 100:15) at RT for 5 min. Finally, the sections were washed and dehydrated with a series of ethanol and xylene (water, 70 %, 90 %, 100 % ethanol and 100 % xylene) and mounted with EUKIT (Asone).

### Immunofluorescence staining

Immunofluorescence staining was performed as previously reported [23]. Primary antibodies, anti-Myosin heavy chain (Developmental Studies Hybridoma Bank, MF20), anti-phosphorylated Histone H3 (Millipore, 06–570) and anti-active Caspase 3 (BD Pharmingen, 559565), were used at dilutions of 1:100 (vol/vol). Secondary antibodies, Alexa 488-conjugated anti-mouse and anti-rabbit IgG and Alexa 594-conjugated rabbit IgG (Molecular Probes), were used at dilutions of 1:400. The procedure for muscle identification in *X. tropicalis* was adapted from that previously reported for *Eleutherodactylus coqui* [25].

### *In situ* hybridization

Complete coding regions of each gene (*shh*, *sox9*, and *irx1*) were amplified from the cDNA prepared from stage 30 *X. tropicalis* embryos and cloned into pcDNA3 (Invitrogen), and DIG-RNA probes were synthesized by SP6 RNA polymerase (Roche, 810274). The probes were denatured at 80 °C for 5 min and then immediately cooled and stored at −30 °C until use. The hindlimb buds were fixed with MEMFA (0.1 M MOPS pH 7.4, 2 mM EGTA, 1 mM MgSO_4_, 3.7 % formaldehyde) at RT overnight. Limb buds were washed with PBT (0.1 % tween/PBS), 25 % ethanol/PBT, 50 % ethanol/PBT, 75 % ethanol/PBT, and 100 % ethanol (two times) each for 5 min at RT and were then preserved at −80 °C. Limb buds were rehydrated with ethanol/PBT series and permeated with 30 % sucrose/PBT until the samples sank. They were embedded in OCT compound (Sakura Fine Tech, 4583) and preserved at −80 °C. Frozen blocks were sectioned into 10-μm-thick samples and subjected to *in situ* hybridization as previously described [23]. The primer sequences used for cloning are shown below.

*Xt shh* forward (BamHI): CGCGGATCCATGCTGGTTGTGACTCGAATTCTGC

*Xt shh* reverse (XhoI): CCGCTCGAGTCAACGGATTTCGTTGCCGCCATG

*Xt sox9* forward (HindIII): CCCAAGCTTATGAATTCTTGGATCCCTTCATGA

*Xt sox9* reverse (XhoI): CCGCTCGAGCTAGGGCCTGGTGAGCTGTGTATAG

*Xt irx1* forward (HindIII): CCCAAGCTTATGTCCTTCCCTCAGCTGGGCTACC

*Xt irx1* reverse (XhoI): CCGCTCGAGTCAGGCAGAGGGAAGTGCTGTCAAT

## Results and discussion

**Fig. 1:**
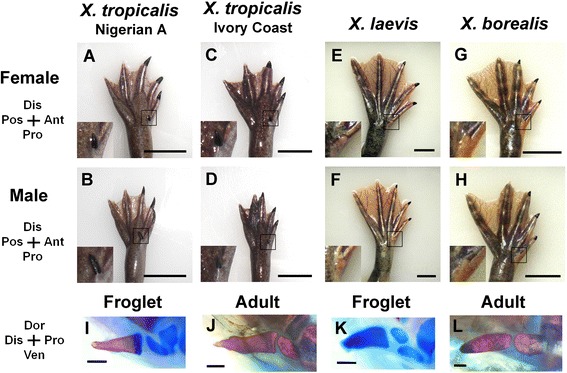
Claw-associated protrusion in *Xenopus tropicalis*. **a, b,** Feet of an adult *X. tropicalis* (Nigerian A line) female (**a**) and male (**b**) with the claw-associated protrusion (inset). **c, d,** Feet of an *X. tropicalis* (Ivory Coast line) female (**c**) and male (**d**) that exhibit a clawed protrusion (inset). **e–h,** Feet of an adult *X. laevis* (**e, f**) and *X. borealis* (**g, h**) female and male with a tiny clawless protrusion (inset). Scale bar in a–h: 10 mm. Images are in ventral view in a–h. **i–l,** Bone and cartilage staining of the protrusion in *X. tropicalis* froglet (**i**) and adult (**j**) and the prehallux in *X. laevis* froglet (**k**) and adult (**l**). Scale bars: 200 μm in i, k and 1 mm in j, l. Images are in anterior view in i–l

We first observed that a line of *X. tropicalis*, Nigerian A [26], has an anterior-most clawed protrusion on the foot (insets in Fig. 1a, b) in addition to clawed digits I, II, III and non-clawed digits IV and V. The clawed protrusion was seen on the feet of both females and males. Another genetically distinct population of *X. tropicalis*, the Ivory Coast line [26], also exhibits an anterior-most clawed protrusion (Fig. 1c, d). The existence of this non-biased phenotype in both sexes and two independent strains suggests that the anterior-most clawed protrusion is a species-specific trait of *X. tropicalis*.

The position of the clawed protrusion in *X. tropicalis* corresponds to that of the prehallux, which is present in the region anterior to the joint between the anterior-most metatarsal and tarsal in other anurans. In the adult *X. tropicalis* hindlimb, the protrusion is not located in the same plane as digits (Fig. 1a-d), and it is located and oriented ventrally on the plantar surface of the foot. Careful examination of the same region in *X. laevis* (Fig. 1e, f) and *X. borealis* (Fig. 1g, h) revealed the presence of a small arch that lacks a claw. Whether the prehallux indeed represents a rudimentary digit or is a tarsal element has been controversial, and identification of this structure has been based on observation of bony or cartilaginous elements in many anuran species [5, 20]. In the *X. laevis* froglet, we found that the clawless protrusion was composed of three non-ossified cartilaginous elements (Fig. 1k), but there were two clearly ossified skeletal elements in the mature adult frog (Fig. 1l). The clawed protrusion in *X. tropicalis* contained two elements, the distal one of which was ossified in the froglet (Fig. 1i, Additional file 1: Fig. S1G), but the proximal element was also eventually ossified in the adult (Fig. 1j).

**Fig. 2:**
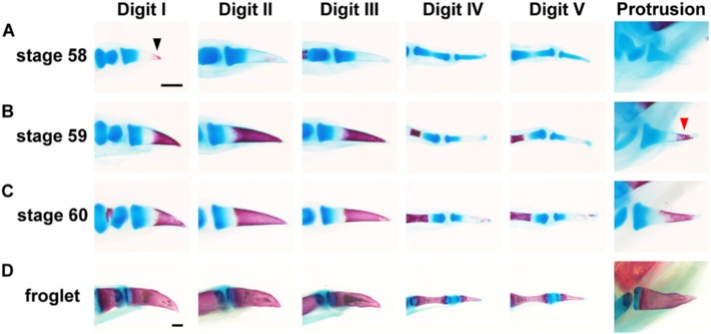
Ossification of distal phalanges and the sixth protrusion in *X. tropicalis.* **a–d**, Bone and cartilage at the distal-most region in the *X. tropicalis* hindlimb. **a**, Stage 58. Ossification of the distal phalanx in digit I was detectable (shown by black arrowhead). **b**, Stage 59. Substantial ossification of the distal phalanges of digits I, II and III could be seen. Ossification of the distal element in the sixth protrusion was initiated (shown by red arrowhead). **c**, Stage 60. Ossification of the distal phalanges in digits IV and V started to be visible. Note that in all distal elements, ossification is initiated at the distal ends of the structures. **d**, The froglet showed complete ossification of the distal elements in all of digits I–V and the protrusion. Images are anterior views. Proximal is left and dorsal is top. Scale bars: 200 μm

To examine the ossification mode (timing and location) of the distal phalanges and the sixth protrusion in the *X. tropicalis* hindlimb, we stained and observed cartilage and bone in the hindlimb during metamorphosis. We detected that the digit in which the distal phalanx first started to undergo ossification was digit I, and the ossified region could be seen at the apex of the distal phalanx at stage 58 (arrowhead in Fig. 2a) [22]. The ossified region with a conical shape at the apex of the distal phalanx in digit I expanded in the proximal direction as metamorphosis proceeded (Fig. 2b, c), and most of the distal phalanx of digit I had ossified by the froglet stage (Fig. 2d). This direction of ossification in the distal phalanx of digit I was similar to that in other digits, and the onset of ossification of the distal phalanx was earliest in digit I, followed by digits II and III, and the distal phalanges in digits IV and V finally started to ossify at stage 60. The distal element in the sixth protrusion started to ossify at stage 59 (one stage later than digit I) (red arrowhead in Fig. 2b), and the ossification mode was very similar to that in the distal phalanges. The above observations indicate that the distal skeletal element of the sixth protrusion resembles the distal phalanx in final morphology and ossification mode.

**Fig. 3:**
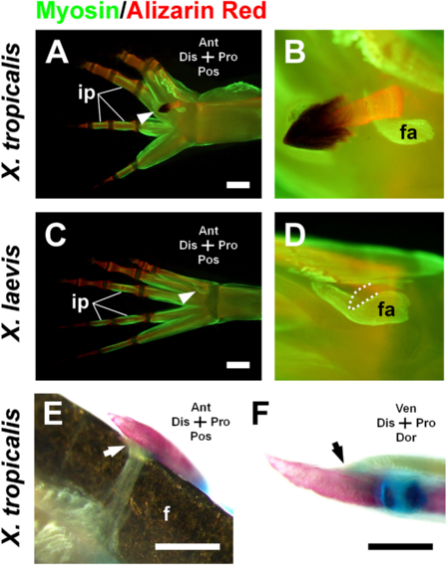
Topological relation of the protrusion with muscle and tendon. **a–d**, Muscles and bones in the hindlimb feet of *X. tropicalis* (**a, b**) and *X. laevis* (**c, d**). Arrowheads indicate the protrusion (**a**) and prehallux (**c**). ip: *interphalangei*, fa: *flexor accessories*. (**b**) and (**d**) are high magnification images of those in (**a**) and (**c**), respectively. **e, f**, A tendon was connected to the distal protrusion of *X. tropicalis* (**e**) and the distal phalanx (digit III) (**f**) (arrows). f: forceps for highlighting the tendon. Images in (**a**–**e**) are ventral views, and (**f**) is a posterior view. Scale bars: 1 mm in (**a, c**) and 500 μm in (**e**, **f**)

We next examined the topological relation of muscle and tendon with the skeletal element of the sixth protrusion. In the hindlimb digits in anurans, the proximal phalanges are associated with muscles (*interphalangei*), but the distal one or two phalanges have no direct muscle association (Fig. 3a, c). The distal ossified element in the sixth protrusion of *X. tropicalis* exhibited no direct association with muscle (Fig. 3a, b. fa in Fig. 3b is *flexor accessories* that exists independently of and behind the protrusion.). The prehallux of *X. laevis* also showed no direct association with muscles (Fig. 3c, d). The distal phalanx in a digit is directly connected to a tendon (black arrow in Fig. 3f), and the distal element in the sixth protrusion of *X. tropicalis* is also connected to a tendon (white arrow in Fig. 3e). Taken together, these findings demonstrate that the distal ossified element of the sixth protrusion is anatomically similar to the distal phalanx.

**Fig. 4:**
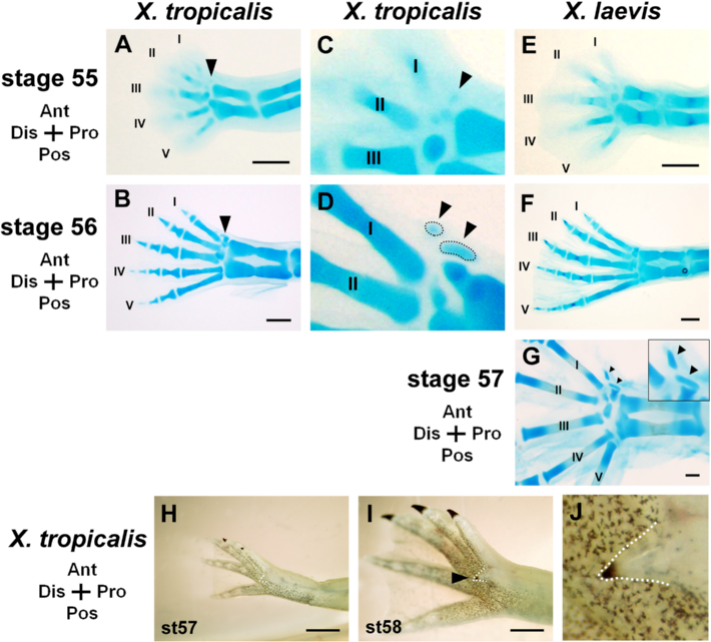
Development of digits and the sixth protrusion in *X. tropicalis.* **a–d**, Digit development in the *X. tropicalis* hindlimb*.* **a, c**, Cartilaginous primordium of the sixth protrusion was detected at stage 55 (shown by arrowhead). **b, d**, Two elements of the protrusion were detected at stage 56. (**c**) and (**d**) are high magnification images of those in (**a**) and (**b**). Arrowheads and dotted lines indicate cartilaginous elements of the protrusion. **e, f**, Development of digits and the prehallux in the *X. laevis* hindlimb. At stages 55 and 56, cartilaginous condensation for the prehallux was undetectable. **g**, Condensation began at stage 57. **h–j**, External appearance of the protrusion at stage 57 (**h**) and stage 58 (**i, j**). **j**, A high magnification image of the protrusion in (**i**) that shows a clear, pigmented claw. All images are ventral views. Scale bars: 250 μm in (**a**, **b**, **e–g**) and 1 mm in (**h, i**)

The findings that the sixth protrusion in *X. tropicalis* contains a claw and ossified elements, and that it has a direct association with a tendon strongly suggest that it represents a digit. To confirm the structure’s identity, we undertook an analysis of its early development. In *X. tropicalis*, in addition to cartilaginous elements in posterior digits II–V and a small cartilaginous element representing digit I (Fig. 4a), we first found at stage 55 that a cartilaginous element appeared at the anterior and proximal region to the metatarsal of digit I (arrowhead in Fig. 4a, c and Additional file 1: Fig. S1A). At stage 56, the distal element was first visible at the prospective protrusion (Fig. 4b, d), and the two elements were clearly detected at stage 57 (Additional file 1: Fig. S1B). Thus, the sixth protrusion in *X. tropicalis* developed soon after digit I formation began, aligned with other digits. The sixth protrusion formed in the same plane as the other digits (Fig. 4b, d). The sixth digit subsequently began bending ventrally (Fig. 4h, i) and resulted in the final ventral location in the adult autopod (Fig. 1a-d). Cornification and keratinization of the sixth protrusion in *X. tropicalis*, as indicated by pigment deposition at the distal tip of the protrusion, were initiated at stage 58 (Fig. 4i, j), and the pigmentation of the sixth protrusion followed that of digit II (Fig. 4h-j). Together, the sixth protrusion develops as the last one in the same manner as that of the other digits. In *X. laevis,* no additional elements were observed at stages 55–56 (Fig. 4e, f), but two prehallux elements were seen at later stages (Fig. 4g, and see also Additional file 1: Fig. S1E, F). In both *X. tropicalis* and *X. laevis*, there were no detectable differences in cell proliferation and apoptosis between digit condensations of digits I–V and the region corresponding to the sixth protrusion (Additional file 1: Fig. S2), suggesting that outgrowth of the sixth protrusion is not due to an extraordinary change in cell proliferation and death.

**Fig. 5:**
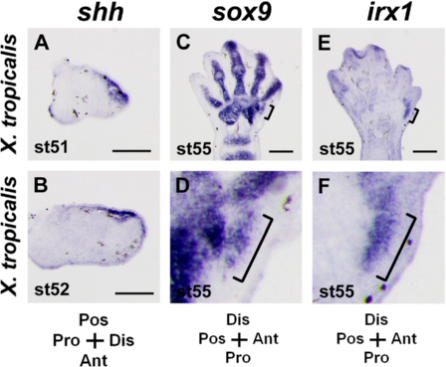
Molecular marker expression in the developing hindlimb of *X. tropicalis*. **a-f**, Gene expression detected by section *in situ* hybridization. **a, b**, Expression of *shh* at stages 51 (**a**) and 52 (**b**). **c**, Expression of *sox9* at stage 55. **e**, Expression of *irx1* at stage 55. Cartilaginous condensation for the anlage of the protrusion (bracket) was *irx1*-posititve. **d, f**, High-magnification images of those in (**c**) and (**e**). Note that *irx1* was highly expressed in the anlage of the protrusion (bracket in **f**). Cartilaginous primordium of the protrusion is indicated by the bracket. Scale bars: 200 μm

To investigate the molecular mechanism of the formation of the sixth protrusion, we examined the expression pattern of some key genes in the developing hindlimb of *X. tropicalis*. Expression of *sonic hedgehog* (*shh)*, a determinant of posterior digit identities, was not detected in the anterior region of the limb bud (Fig. 5a, b), suggesting that the development of the sixth protrusion is not due to unnatural ectopic expression of *shh*, unlike many cases of polydactyly in other animals [27–29]. Expression of *sox9*, a marker of precartilaginous condensation, was detected in the region destined to form the sixth protrusion at stage 55 (Fig. 5c, d), just prior to cartilage differentiation (Fig. 4a, c). We elucidated a molecular feature of the precartilaginous anlage of the sixth protrusion by using a digit condensation marker, *irx1*, that is known to be exclusively expressed in the digit anlagen during the early phase of digit formation in the mouse, chicken [30–32] and *Xenopus* (Additional file 1: Fig. S3). The anlage of the sixth protrusion clearly expressed *irx1* (Fig. 5e, f), indicating that the sixth protrusion has characteristics of the acropodium (phalanges and metatarsals) but not of the mesopodium (tarsals) that forms highly diversified elements. Interestingly, we obtained no evidence of transition from a tarsal anlage to a digit-like structure during development, and the primordium of the sixth protrusion developed as a digit from the beginning. In spite of its small and less-segmented morphology, the protrusion can stand comparison with other digits.

From the above analyses and observations of the gross morphology, tissue composition and molecular features of the anterior-most sixth protrusion in the *X. tropicalis* hindlimb*,* we propose that this structure is a true digit. If this is the case, it indicates that the pentadactyl constraint cannot be applicable to all living tetrapods (at least to this species), and we should reconsider the concept of a pentadactyl ground state. The individual digits of hands and feet with five and fewer digits are understood to exhibit separate identities, based on the different numbers of phalanges, as well as their different sizes and morphologies [6]. Notably, there are only two ossified elements of the sixth digit in *X. tropicalis*, and we classified the distal and proximal ones as the phalanx and metatarsal elements, respectively. Therefore, we consider the phalanx numbers of digits in the hindlimb of *X. tropicalis* to be 1, 2, 2, 3, 4, and 3 in digits 0, I, II, III, IV, and V, respectively (We did not re-number the digits, but instead named the sixth digit “digit 0”.).

Once the sixth protrusion in *X. tropicalis* is identified as a digit, the nature of the prehallux in other species of the *Xenopus* genus can be re-assessed. The prehallux in other *Xenopus* species (*X. laevis* and *X. borealis*) occupies the same position as the sixth digit in *X. tropicalis* and has a similar skeletal morphology (Fig. 1). Thus, the prehallux in *X. laevis* and *X. borealis* morphologically and topologically resembles the sixth digit in *X. tropicalis*, although the structure in *X. laevis* and *X. borealis* is smaller than that in *X. tropicalis*. We propose that the prehallux in these species also represents a rudimentary digit. *X. laevis* and *X. borealis* form a pipid clade [33–35], which seems to be a derivative taxon with genome duplication, and reduction of the digit may have proceeded in this lineage. There has been long debate on whether the prehallux of anurans represents a digit or not [5, 20], and our proposal corresponds to the argument that the prehallux of anurans represents a rudimentary digit [5, 20]. We also agree with the comment in [5] that “the confusion about whether the prepollex and prehallux are digits seems to be caused mainly by the once-powerful paradigm of an archetypal tetrapod hand and foot with five digits”. Indeed, the prehallux in anuran limbs shows highly diversified morphologies, although the prehallux is usually much smaller than digits I-V [20]. We surveyed literature that described the prehallux in anuran species [17, 20, 34, 36–41] and compared phylogenetic relationships. Although the number of reports we refer to is limited, they show that throughout anuran phylogeny, there is a wide range of groups that include species with the prehallux, suggesting that the sixth digit (digit 0/prehallux) has been conserved among extant anurans [20]. Urodeles, another major clade of lissamphibians, generally have hindlimbs with five digits. Despite the evolutionary tendency for reduced skeletal elements in their hindlimb extremities, some urodele species have a prehallux [42], as demonstrated by *Ranodon sibiricus,* an extant salamander in which a sixth digit-like structure is visible at the larval stage (Additional file 1: Fig. S4), [38]. Thus, the argument that the prehallux is a rudimentary digit in anurans could be applied to urodeles. Extant amphibians are thought to have evolved from ancestors that have five digits [16]. Since polydactyly has been assumed to have preceded pentadactyly, it is possible that the sixth digit in extant amphibians has re-appeared and represents an atavistic structure in extant amphibians. The developmental mechanism of the sixth digit formation in *X. tropicalis* might reflect the ancestral condition of polydactyl digit formation.

Another hypothesis, which currently appears much less likely, is that the sixth digit persisted, undetected, throughout amphibian evolution. This hypothesis is not supported by the current understanding of the fossil record [16, 43] and would require reinterpretation of many fossils. However, given the small size of the sixth digit, it remains possible that it went unnoticed in many taxa, given that paleontologists did not expect to see it. One possible example of a fossil that could be re-examined in the light of our findings was described by DeMar (1968: Figure 17 [44]). It seems possible to interpret that foot as having had six digits, even though DeMar (1968, [44]) did not appear to have considered this possibility. This hypothesis would also require finding the sixth digit in a large number of urodele and anuran species, which is not clearly documented at present. However, we hope that raising the possibility that a sixth digit has persisted throughout amphibian evolution will prompt a re-examination of digit number in extant and extinct amphibians.

To the best of our knowledge, there is no report of a sixth digit in extant tetrapod species in nature, and only rare cases of extinct species such as *Nanchangosauridae* [45] and *Ichthyosaurus* [46]. They are very rare cases compared with all of the extinct and extant tetrapod species in which the digit number was maintained or reduced during evolution. There are many polydactyly mutant model animals in laboratories [11, 27, 29, 47–53] or domestic animals [54–56] on farms that are protected by humans from natural selection. Polydactyl individuals also occasionally appear [4, 57, 58], but polydactyly mutation is not usually stabilized in species. The hypothesis that the sixth digit of *Xenopus tropicalis* is a result of increased digit number should be further substantiated.

## Additional file

Additional file 1: Figure S1. Histological analysis of the protrusion of *X. tropicalis*. A-D, Alcian blue staining and Elastica van Gieson staining of the protrusion during metamorphosis of *X. tropicalis* at stages 55+ (A), 57 (B), 58 (C) and 62 (D). **E**, **F**, Elastica van Gieson staining of the prehallux of *X. laevis* at stages 55+ (E) and 57 (F). At stage 55+, the proximal element of the protrusion was detected in *X. tropicalis* but not in *X. laevis.* **G**, 3D reconstruction of ossified elements in the hindlimb of *X. tropicalis* at froglet stage. Dotted lines indicate cartilaginous elements. t: tibiale, Y: element Y, p: proximal element of the protrusion, d: distal element of the protrusion. Scale bar: 100 μm. **Figure S2.** Cell proliferation and apoptosis in the hindlimb of *X. tropicalis *and *X. laevis*. Developing limbs were immunostained for cell proliferation (phosphorylated Histone H3) and apoptosis (active Caspase 3). **A-D,** Distributions of phosphorylated Histone H3-positive cells in stage 54 and stage 55 limb buds of *X. tropicalis* (**A**, **B**) and *X. laevis* (**C, D**). Cell proliferation was evident throughout the limb bud. **E-H,** Distributions of active Caspase 3-positive cells in stage 54 and stage 55 limb buds of *X. tropicalis* (**E**, **F**) and *X. laevis* (**G, H**). Physiological apoptosis was enriched in the apical ectodermal ridge (AER). Note that no detectable difference in cell proliferation or apoptosis was observed between *X. tropicalis* and *X. laevis* at these stages. Arrowheads indicate regions where the prehallux anlage is formed. Images are ventral views. Distal is left and anterior is top. Scale bars: 200 μm. **Figure S3.** ***irx1*** **expression specific for digit condensation of the** ***X. tropicalis*** **hindlimb bud. A**, *sox9* expression at stage 53. Precartilaginous condensation, including the first visible condensation of digit IV, was detectable. **B**, *irx1* expression at stage 53. *irx1* was exclusively expressed in the digit condensation of digit IV at this stage, and other cartilaginous regions were *irx1*-negative, indicating that *irx1* expression is specific for developing digits. Scale bar: 200 μm. **Figure S4. Skeletal drawings of** ***X. tropicalis*** **and** ***Ranodon (R.) sibiricus*** **.** Left: Outline drawing of skeletal components in the hindlimb of an *X. tropicalis* froglet. The sixth protrusion is named digit VI. Right: Outline drawing of skeletal components in the hindlimb of an *R. sibiricus* larva (modified from Vorobyeva, 2014) [35]. It shows the digital anlagen of the sixth digit (we considered it as digit 0) at the larval stage.
