# Supplementary material for: Evidence for an amphibian sixth digit
Source: Zoological Lett. 2015 Jun 15;1:17. doi: 10.1186/s40851-015-0019-y (PMC4657212; doi:10.1186/s40851-015-0019-y)
Supplement: Additional file 1: Figure S1. — Histological analysis of the protrusion of X. tropicalis. A-D, Alcian blue staining and Elastica van Gieson staining of the protrusion during metamorphosis of X. tropicalis at stages 55+ (A), 57 (B), 58 (C) and 62 (D). E, F, Elastica van Gieson staining of the prehallux of X. laevis at stages 55+ (E) and 57 (F). At stage 55+, the proximal element of the protrusion was detected in X. tropicalis but not in X. laevis. G, 3D reconstruction of ossified elements in the hindlimb of X. tropicalis at froglet stage. Dotted lines indicate cartilaginous elements. t: tibiale, Y: element Y, p: proximal element of the protrusion, d: distal element of the protrusion. Scale bar: 100 μm. Figure S2. Cell proliferation and apoptosis in the hindlimb of X. tropicalis and X. laevis. Developing limbs were immunostained for cell proliferation (phosphorylated Histone H3) and apoptosis (active Caspase 3). A-D, Distributions of phosphorylated Histone H3-positive cells in stage 54 and stage 55 limb buds of X. tropicalis (A, B) and X. laevis (C, D). Cell proliferation was evident throughout the limb bud. E-H, Distributions of active Caspase 3-positive cells in stage 54 and stage 55 limb buds of X. tropicalis (E, F) and X. laevis (G, H). Physiological apoptosis was enriched in the apical ectodermal ridge (AER). Note that no detectable difference in cell proliferation or apoptosis was observed between X. tropicalis and X. laevis at these stages. Arrowheads indicate regions where the prehallux anlage is formed. Images are ventral views. Distal is left and anterior is top. Scale bars: 200 μm. Figure S3. irx1 expression specific for digit condensation of the X. tropicalis hindlimb bud. A, sox9 expression at stage 53. Precartilaginous condensation, including the first visible condensation of digit IV, was detectable. B, irx1 expression at stage 53. irx1 was exclusively expressed in the digit condensation of digit IV at this stage, and other cartilaginous regions were irx1-negative, indicating that irx [file 40851_2015_19_MOESM1_ESM.pdf]

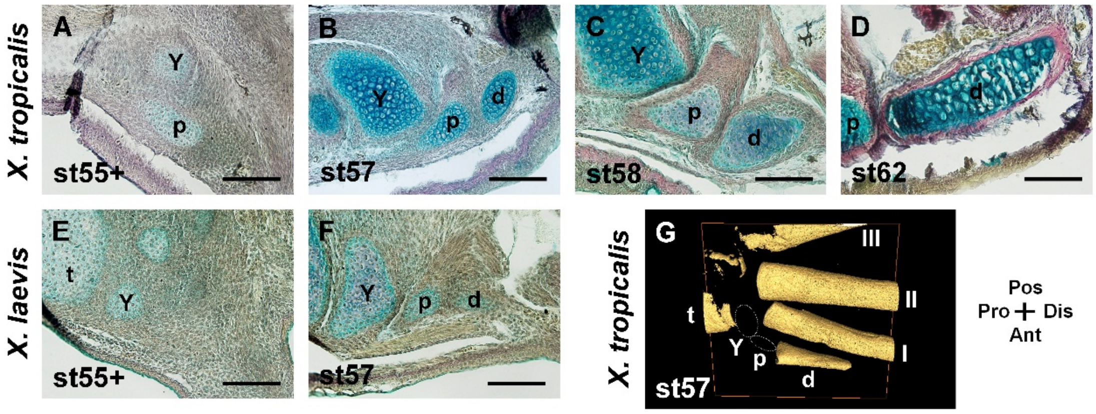

Figure S1. **Histological analysis of the protrusion of *X. tropicalis.***

A-D, Alcian blue staining and Elastica van Gieson staining of the protrusion during metamorphosis of *X. tropicalis* at stages 55+ (A), 57 (B), 58 (C) and 62 (D). **E, F,** Elastica van Gieson staining of the prehallux of *X. laevis* at stages 55+ (E) and 57 (F). At stage 55+, the proximal element of the protrusion was detected in *X. tropicalis* but not in *X. laevis*. **G**, 3D reconstruction of ossified elements in the hindlimb of *X. tropicalis* at froglet stage. Dotted lines indicate cartilaginous elements. t: tibiale, Y: element Y, p: proximal element of the protrusion, d: distal element of the protrusion. Scale bar: 100 µm.

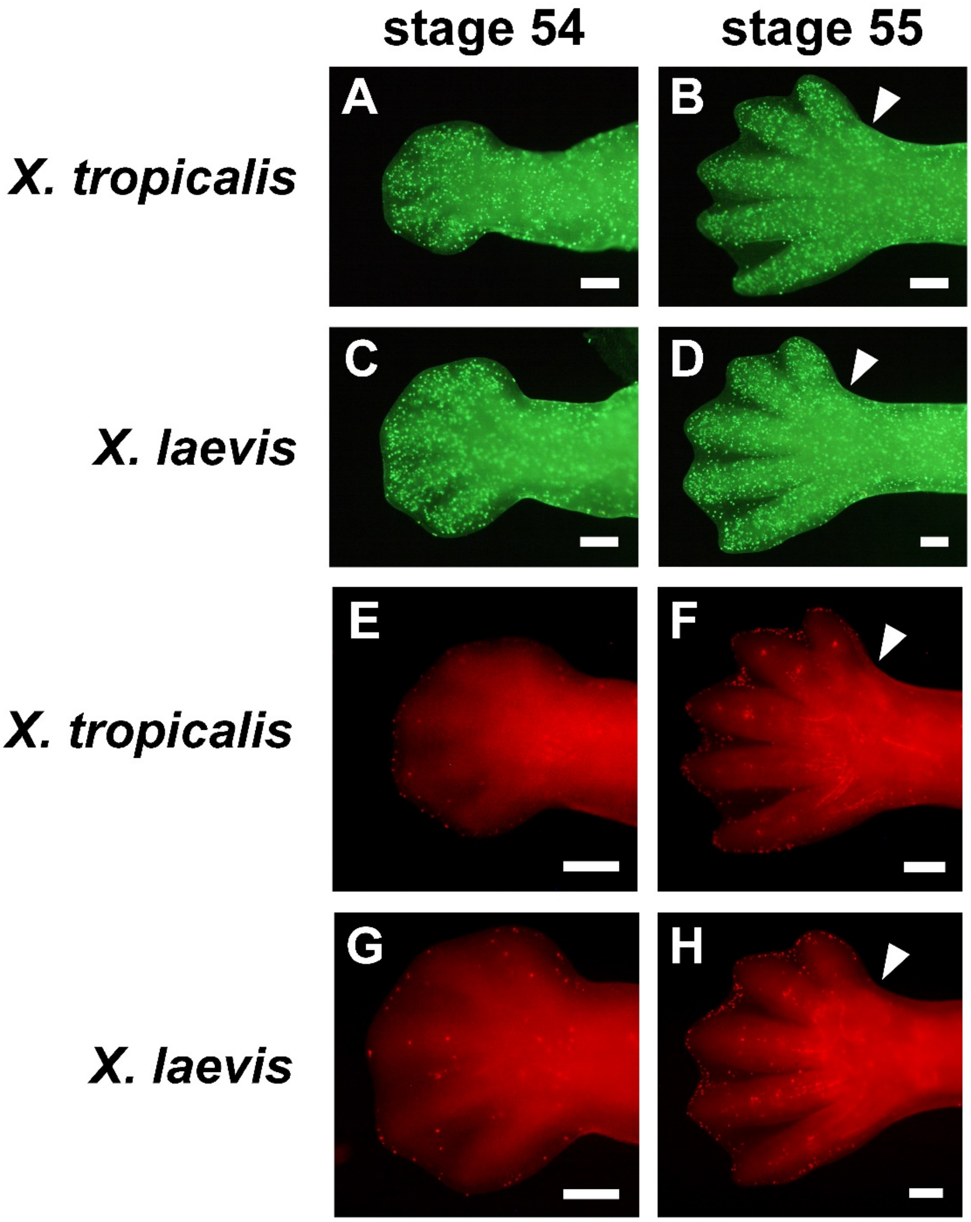

Figure S2. Cell proliferation and apoptosis in the hindlimb of *X. tropicalis* and *X. laevis*. Developing limbs were immunostained for cell proliferation (phosphorylated Histone H3) and apoptosis (active Caspase 3). **A-D,** Distributions of phosphorylated Histone H3-positive cells in stage 54 and stage 55 limb buds of *X. tropicalis* (**A**, **B**) and *X. laevis* (**C, D**). Cell proliferation was evident throughout the limb bud. **E-H,** Distributions of active Caspase 3-positive cells in stage 54 and stage 55 limb buds of *X. tropicalis* (**E**, **F**) and *X. laevis* (**G, H**). Physiological apoptosis was enriched in the apical ectodermal ridge (AER). Note that no detectable difference in cell proliferation or apoptosis was observed between *X. tropicalis* and *X. laevis* at these stages. Arrowheads indicate regions where the prehallux anlage is formed. Images are ventral views. Distal is left and anterior is top. Scale bars: 200 µm.

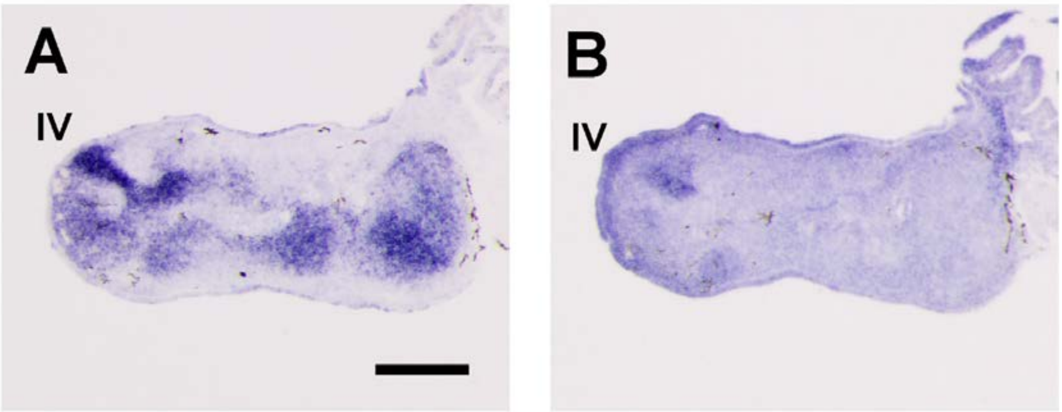

Figure S3. ***irx1*** **expression specific for digit condensation of the** ***X. tropicalis*** **hindlimb bud.**

A, *sox9* expression at stage 53. Precartilaginous condensation, including the first visible condensation of digit IV, was detectable. **B**, *irx1* expression at stage 53. *irx1* was exclusively expressed in the digit condensation of digit IV at this stage, and other cartilaginous regions were *irx1*-negative, indicating that *irx1* expression is specific for developing digits. Scale bar: 200 µm.

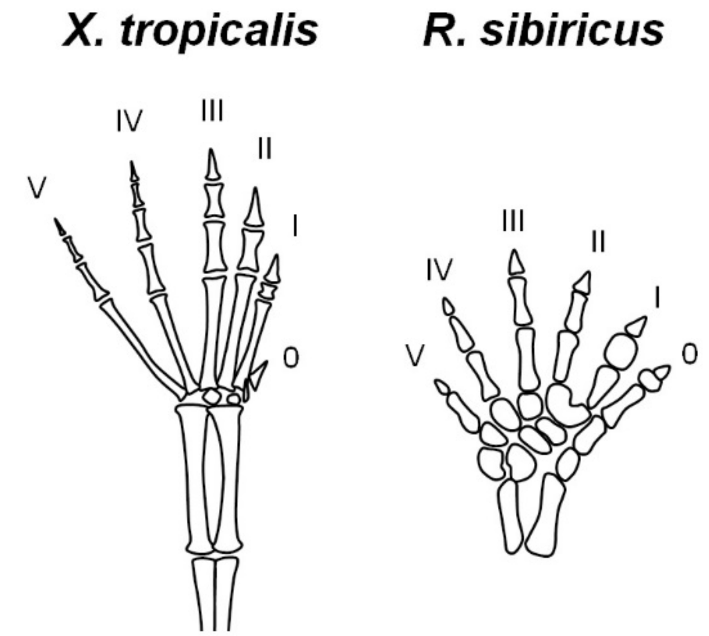

Figure S4. Skeletal drawings of *X. tropicalis* and *Ranodon (R.) sibiricus*.

Left: Outline drawing of skeletal components in the hindlimb of an *X. tropicalis* froglet. The sixth protrusion is named digit VI. Right: Outline drawing of skeletal components in the hindlimb of an *R. sibiricus* larva (modified from Vorobyeva, 2014)[35]. It shows the digital anlagen of the sixth digit (we considered it as digit 0) at the larval stage.
